# From conventional healthcare to e-health: Social and spatial transformation. Using a comparison between Hong Kong and Mainland China

**Published:** 2021-09-29

**Authors:** Carine Milcent

**Affiliations:** Department of Economics, French National Centre for Scientific Research, Paris School of Economics, Paris, France

**Keywords:** healthcare, China, spatial dimension, digital healthcare

## Abstract

**Background and Aim:**

Does spatial organization of healthcare access still matter in China? I assess how e-health has transformed the notion of healthcare access and what spatial dimension of healthcare means in China today and in the near future. I also consider a dynamic perspective to propose keys to anticipate upcoming changes. Hong Kong has a very efficient healthcare system with a dense network of high-level hospitals and a high level of healthcare access. In major Chinese urban centers, a deliberate policy to improve healthcare availability has resulted in a spectacular increase in the number of healthcare structures over the last two decades. This includes urban healthcare centers and outpatient clinics. Nevertheless, the most of the population faces explicit and implicit financial penalties to get full healthcare access. To solve the problem, a digital health revolution is emerging.

**Methods:**

I use a qualitative case study approach. I conducted a series of semi-structured, face-to-face interviews to evaluate experiences, expectations, and opinions of patients regarding healthcare access and its associated financial burden as well as their use of digital health.

**Results:**

I assessed how e-health has transformed the notion of healthcare access and what spatial dimension of healthcare means today and in the near future in China. I also considered a dynamic perspective to propose keys to anticipate upcoming changes. Healthcare centers tend to shift from a place to get cured to a link within an e-healthcare pathway. For instance, this is a place to get e-prescribed medication. Advantages of this shift include a reduction in the cost of healthcare and remote access to highly qualified medical staff, bypassing the lack of trust in the quality of care offered in local hospitals.

**Conclusion:**

A forward-looking approach suggests that e-healthcare is becoming the entry point to healthcare for a large part of the population.

**Relevance to Patients:**

This study informs the policy makers of upcoming changes, and contributes to understanding and anticipating modifications needed in the healthcare system.

## 1. Introduction

Does spatial organization of healthcare access still matter in China? To address this question, it is worth comparing the situation in Hong Kong and in major urban centers in Mainland China.

Hong Kong has a very efficient healthcare system, classified as one of the best.^1^According to the United Nations, the territory has the highest life expectancy in the world.^2^

There is only scarce literature comparing Hong Kong with Mainland China in terms of healthcare access. Kong *et al*. [1] give an overview of medical and health services in Hong Kong, emphasizing the place of the central administration for hospital management. Fitzner *et al*. [2] present the Mainland Chinese healthcare system, associated with its economic foundations, from a centrally planned economy to a market economy. Zhang and Wang [3] analyze the health management system model. In Mainland China, two main public authorities manage public hospitals: The National Health and Family Planning Commission (NHFPC) and health bureaus at the local government level. According to Kong *et al*. [1], having two heads is an “obvious disadvantage of China’s medical system.” In 1990, a new health administration system was introduced in Hong Kong, and since 1991, the Hospital Authority runs the management of all public hospitals. “It directly establishes, manages, controls, and develops public hospital systems.” The authors describe it as the key to an efficient organization. However, this paper does not provide a description of each system. It also does not present the e-health development and the spatial dimension of the two systems. In this paper, not only do I present the two systems but I also focus on the role of e-health, taking into account the spatial dimension of healthcare access.

For 7.2 million urban people over 420 square miles of land, there are 42 public and 11 private hospitals, which make physical access to healthcare providers easy in Hong Kong. The healthcare system is based on a Beveridge model of universal access to secondary healthcare services, with many similarities to the National Health Service (NHS) in the UK. On the other hand, primary healthcare, for which there is no public insurance, is mainly provided by private entities, making the situation more comparable with the US system. To get information on hospital activity, there is a unified medical information system. The Electronic Medical Record (EMR) System has been implemented for information sharing between the public and private sectors in Hong Kong. An array of papers investigates the adoption and efficiency of the EMR System that is now used in all Hong Kong public hospitals [4-8]. These papers assess how the information is distributed throughout the health profession, and the efficiency of such information technology in medical practice. In this paper, the EMR is presented to illustrate the difference between deployment of the healthcare information system implemented in Hong Kong and the offline healthcare provided to the population as compared to the online healthcare provided in Mainland China, with its weaker healthcare information system.

In Mainland China, the healthcare system covers a variety of contexts. Public hospitals in first-tier cities^3^ are quantitatively and qualitatively different from those in lower-tier cities. In addition, inter-city differences and even inter-province differences exist. Comparing Hong Kong and Mainland China in its entirety is not possible. Mainland China is too vast and contains too many possibilities of healthcare access. However, contrasting Hong Kong with major Chinese urban centers makes sense in a social and spatial transformation perspective.

For these areas, there are very obvious inefficiencies in the health system. Some examples: the increasing number of patients opting to go to high level or city hospitals, leading to the transfer of resources to them; overused and expensive drugs. Despite amazingly comprehensive efforts undertaken by a rapidly changing healthcare system, persistent challenges remain [9].

The market drives healthcare system access. The different levels of government (from the central, state, and county authorities) barely finance public hospitals. In spite of the implementation of a zero mark-up policy on drugs to limit over-prescription and the ongoing development of public health insurance schemes, both public and private healthcare remain financially inaccessible to a large part of the population. There is a large amount of literature on the healthcare system, its inefficiencies, the series of reforms, and the place of digital healthcare [9-14].^4^

These two situations in Hong Kong and in some large cities in Mainland China should not be seen from a static point of view. Considering a dynamic perspective is a key to anticipating upcoming changes. The emergency department is used as an entry point for primary care services in hospitals, and a shortage of health professionals is another common crucial issue. Health empowerment, as well as the rapid spread of digital healthcare tools, is emerging as an alternative solution to ensure accessible, high-quality, and sustainable healthcare.

The spatial dimension is defined by how strong and tight the offline web of healthcare services is. So far, a strong and tight offline web of healthcare services has been a key (but not the only) indicator of an efficient healthcare system. Today, due to e-health, online healthcare providers transform the need for a strong and tight offline web of healthcare services.

The present study aims to scrutinize pre-implementation of generalized online healthcare in China — not only for primary care, but also for secondary care — comparing Hong Kong with some of the largest cities in Mainland China to address the following questions: (i) what is the role of the financial healthcare burden in the adoption of e-health; and (ii) from a dynamic perspective, how does the implementation of a generalized healthcare information system change the need for a strong and tight web of healthcare services?

Using a qualitative case study approach, I assess how e-health has transformed the notion of healthcare access and what spatial dimension of healthcare means in China today and in the near future. I also consider a dynamic perspective to propose keys to anticipate upcoming changes. Then, a forward-looking approach suggests that e-healthcare is becoming the entry point to healthcare for a large part of the population. This study informs the policy makers of upcoming changes, and contributes to understanding and anticipating modifications needed in the healthcare system.

I performed field observations and interviews to understand why online healthcare providers can be preferred to offline healthcare providers. Using Hong Kong and some cities in Mainland China as examples, I compare the context where public hospital admission is free of charge (as in Hong Kong) to that where public hospital admission is a financial burden (as in Mainland China). A qualitative study was conducted using a series of semi-structured, face-to-face interviews to evaluate experiences, expectations, and opinions of patients regarding healthcare access and its associated financial burden. Interview transcripts were content analyzed to identify key factors.

The research question of this paper is organized as follows: in Section 2, I describe the healthcare system in Hong Kong SAR; in Section 3, I present the healthcare system in Mainland China; in Section 4, I propose a dynamic perspective analysis. The conclusion and discussion are in Section 5.

## 2. The Healthcare System in Hong Kong SAR

**Figure 1:**
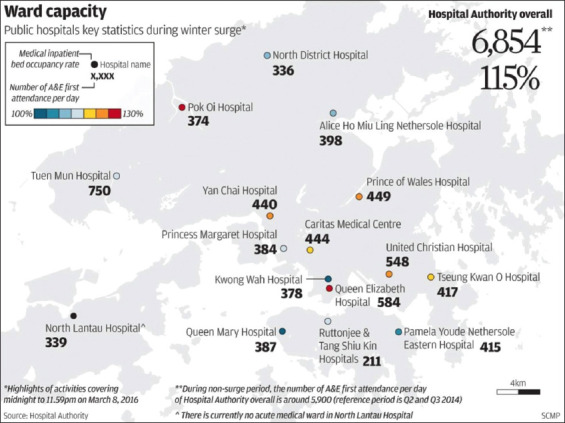
Public hospital web in Hong Kong. Source: http://www.scmp.com/sites/default/files/styles/980w/public/images/methode/2016/03 /10/8e9706d6-e65c-11e5-9c87-1b0e9fb1e112_image_hires.jpg?itok=Wns-7Lbp, published 9 March 2016 (Wednesday).

In Hong Kong, a city of nearly 8 million people, the total amount of health expenditures as percentage of GDP reaches 6.2%, with 3.2% and 3.0% in the public and private sectors, respectively^5^. As illustrated in Figure 1, Hong Kong has a strong and tight web of healthcare services.

The healthcare system in Hong Kong is a dual-track system. The Hospital Authority^6^ manages public healthcare institutes, with 42 public hospitals and 27,895 beds (1,660,000 discharge episodes). It also oversees about 80% of all inpatient services. The quality of services provided in terms of equipment and medical staff training is very high. The patients pay an insignificant fee for it: HK$180 for an admission, regardless of the healthcare service provided and the length of stay. The following example given by Lili^7^, at Princess Margaret Hospital, illustrates this (see Annexe A for details on fees in public hospitals):

“*I have a part-time job with a package that does not include any health insurance package. My husband is a painter artist. He also works as a temporary teacher in a local primary school. We did not plan to have a baby but when we found out it, we were very grateful. At first, I planned to have my delivery at Matilda hospital. Our parents proposed to financially support us. However, the financial burden was too important. We decided to go to a public hospital. We paid only HK$150 for the entire hospital stay and associated medication. Our baby got very good medical treatment. On my part, during the whole process, pregnancy management under medical supervision was highly satisfying*.”

In addition, the public hospital web is very developed. A public hospital is present in each district in Hong Kong. We may argue that the district size, population density, and the socio-economic context differ strongly from one district to another. Furthermore, a more appropriate public hospital organization could be set up. However, it remains true that this hospital organization provides access to a public hospital for any Hong Kong resident.^8^

**Figure 2:**
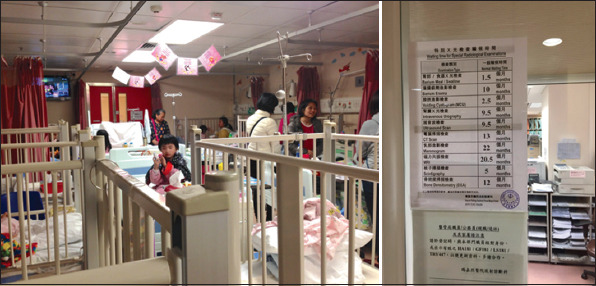
A room in the children’s department and the waiting list displayed in Queen Mary hospital, Hong Kong. Source: Photos taken by the author in March 2016 and February 2016, respectively.

However, in terms of catering and comfort, the patients have to cope with a high number of beds per room. There can be more than 10 beds per room. May, a young girl, had an unknown infection and she had to stay for a few days at Queen Mary hospital (Figure 2). Her mum spent her childhood in Melbourne, Australia. She returned to Hong Kong a decade ago. She tells the story of how many beds were in her daughter’s medical room and how hard it was to spend the nights there.^9^

“*Because of the unusual symptoms of my daughter, we were transferred from the Adventist hospital, a private hospital, to? Queen Mary. First, I felt relieved to see how many staff there were in this private hospital. I also felt relieved that different specialists consulted my daughter. Then, the diagnosis became clearer but some additional medical screening and biological tests were expected. They asked my daughter to stay in hospital to remain under observation for a few days until the final results and a last examination. Here the nightmare started*. [*…*]*She was in a room with more than ten beds. I got a basic chair to remain with her and to rest. The light was turned on almost all night. It was incredibly noisy and uncomfortable. My husband was not permitted to see our daughter due to the visiting hour’s regulation policy*. [*…*] *We left the hospital more tired than when we had been admitted. I promised myself that it would be the last time in such a condition. I highly prefer to stay at home than to bear this hospital’s condition of admission*.”

Larina had a delivery at Queen Mary hospital. She spoke about her experience in this way^10^:

“*I knew that in public hospitals, the medical staff prefers natural delivery without epidural. In case of any complication, they perform a C-section. I wanted to avoid a C-section. However, on the other hand, I could not imagine myself giving birth without epidural anesthesia. The spinal procedure is nothing compared with the pain to give birth. I cried and cried and cried. My husband told me that I was scaring other women. Whatever my pain and my cries, the medical staff never called the doctor. I had to give birth with terrible pain. This is a vivid memory that is linked with public healthcare services in Hong Kong. How a medical staff can be so inhuman with women? For sure, I do not plan to give birth again… and I am motivated to get a job with a very good health insurance package, even if with a lower pay*”

Therefore, even if Hong Kong is often characterized as tantamount to a free market economy, it does not concern the entire economy. Opting for economic liberalism does not necessarily include the healthcare market. It does not mean that private players are excluded from the healthcare market. As I already said, Hong Kong’s healthcare market is definitely a dual-track system. The private sector covers 370,000 discharges in 11 hospitals. Private health charges for the patients are based on a variety of options that tend to the patients’ preferences. The price to pay is high in exchange for luxurious perks.^11^ Non-governmental organizations also have a crucial position. They tend to focus on family health and community health.

The public hospital is not expected to be the access gate to the healthcare system. As a first step, patients have a consultation in the office of a medical professional. Primary care is the common pathway for patients into a continuous healthcare process. In case of a more severe disease, the patient is referred to a hospital. However, if secondary healthcare is mainly publicly provided, primary healthcare care is privately provided. The cost for a primary care consultation can be high. Patients may go to a community health center that can be publicly funded or an NGO health center. However, there are constraints in terms of visiting hours and in availability of specialists.

There is no social health insurance in Hong Kong. The public healthcare system ensures free access to only some healthcare services — the more extreme and expensive ones. The patients are supposed to pay for primary care themselves. The private health insurance (PHI) market is quite well organized and developed. However, this market is often connected to the labor market: depending on the job, the individual or the household benefits from a health insurance package.

As a consequence, the emergency room is used as a gateway to get access to free healthcare services. The free (or for a very marginal cost) service in public hospitals pushes individuals to skip primary care services offered outside of the hospital. They go straight to the hospital emergency department. The result is congestion of public hospitals, not due to healthcare needs, but due to an overuse of the public system. As we see here, the emergency department ensures healthcare access whatever the cost. There is no need for the use of e-healthcare (an e-consultation, for instance) to get affordable access to healthcare at an expected level of quality.

However, another consequence of this behavior is the excessive workload. Beyond the payroll differences between the private sector and the public sector, working conditions are also a determinant as to where medical staffs prefer to do their job [15]. Dealing with a reasonable number of patients definitely eases the workload. In the private to public systems, the ratio of inpatient bed-days is 10:90, inpatient admission is 20:80, and overall outpatient is 70:30 [16]. For the supply side, the workforce ratio of public to private sector is 6:4. Nurses in public hospitals were one for 10 patients, which is much higher than the international standards of one nurse for 5-6 patients [17]. In the same line, 40% of doctors work in public hospitals and they serve 90% of patients. These figures reflect the imbalance of the two sectors in Hong Kong. From a dynamic perspective, it raises the question of the sustainability of such a healthcare system.

## 3. The Healthcare System in Mainland China

The focal point of the difference between Hong Kong and big cities in Mainland China is the network of healthcare institutes and, more specifically, the public hospital network.

In Mainland China, the public hospital is the inner center of the healthcare system. Unlike the situation in Hong Kong, most of the medical staff works in public healthcare institutions. According to the Ministry of Health, 82.8% of all practicing medical doctors work in public healthcare institutions. Moreover, public hospitals provide 89.78% of all outpatient services and 87.92% of all inpatient services.^12^ Aggregate hospital revenue from outpatient services grew from 28 % in 1994 to 48 % in 2018.^13^

Nonetheless, the number of public hospitals is still too small to satisfy demand. Tier I hospitals in big cities of Mainland China have an insufficient healthcare supply system in terms of the spatial dimension of quality required by the demand.

This is still largely the case, in spite of the government’s efforts to change the situation. Over the last two decades, and even more recently, the Chinese government has indeed multiplied actions in favor of healthcare community centers. As early as 1997, the need for the establishment of a primary care network in urban areas has emerged as a priority for the government. Reform-creating community health centers were created in various cities. A series of reforms, largely initiated from 2009, tend to decentralize healthcare suppliers. These reforms aim at offering alternatives to hospitals in the form of other healthcare institutions and then, to extend the healthcare access over the Chinese territory. The stated goal is to change the patient’s pathway. So far, while people view the emergency department of public hospitals as the gateway to healthcare access, the objective is to re-establish primary care outside of the hospital. In 2015, a general practitioner referral system was introduced. The aim is to reduce inappropriate use of higher-tier hospital care. It should have partially solved the congestion issue in public hospitals and re-allocated patients into healthcare facilities best suited to their needs in terms of equipment and qualified medical staff. Yet, this is not what is observed today.

Here, we observe that using the emergency department as a gateway for healthcare services is common in Hong Kong as well as in big cities in Mainland China. However, the determinants differ in some aspects. In Hong Kong, it can be explained by the financial burden of healthcare in any private healthcare center versus the option of a free-of-charge healthcare service in public hospitals. In Mainland China, it is thought to be due to the level of quality offered in high-level public hospitals (called tier I or tier I-AAA) and to the “three-in-one” service in public hospitals not available in the community health centers. Indeed, in public hospitals, the patient gets a preliminary diagnosis from a doctor; then, blood tests, biological procedures, and radiography, as well as any additional medical investigations, are carried out if needed. From the same location, all medical information required to set up adequate and proper treatments is available.

**Figure 3:**
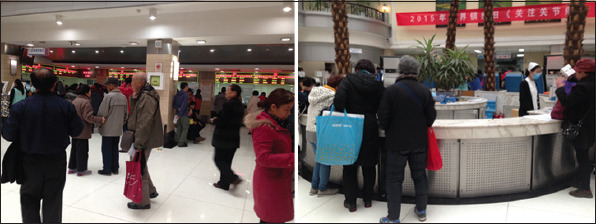
The situation in the Accident and Emergency Department, Hospital of Beijing University, People’s Hospital at 10:00 am on a weekday in November 2015.

We do not have the same issue in Hong Kong because of the healthcare system network. The high density of healthcare institutions allows people to easily access a public hospital when this is required. The spatial dimension of healthcare access is then part of the puzzle of the inefficiency in the Chinese Mainland healthcare system (Figure 3).

Chou, his wife, and their baby boy came from the neighboring Hebei province to Peking Union College Hospital, emergency department. Their baby had a fever and a cough.^14^

“*This is fine, we prefer to be here than in our home. It was too stressful. The medication given by the doctor in the community health center did not work as expected. We are very worried. We did not know the doctor who consulted our baby. He was kind but we are not so sure about the diagnosis. We needed a diagnosis from a more experienced doctor. The night and the following day after the consultation, our baby was still grumpy. We know we have made the right decision*. [*…*] *From our home to the hospital, the trip was 3 hours.”*

This account probably explains the increasing use of e-healthcare services, such as e-consultations, for instance. This is a way to get healthcare at an expected level of quality with neither delay nor extensive travel. In addition, the financial burden is acceptable, even in cases of multiple e-healthcare services.

In Mainland China, the high cost had indeed deterred many families from obtaining necessary healthcare. In response, the Chinese government implemented three programs of public health insurance from the early 2000s for rural residents as well as urban workers and urban residents [18,19]. In 2018, a reform to merge two of these programs, namely the New Cooperative Medical Scheme (NCMS) for rural residents and the Urban Resident Basic Medical Insurance (URBMI) for urban residents, were introduced. However, migrant workers remain insured in their location of origin, in a province different from the province where they live and work. Reimbursement of their cross-province medical expenses needs to be sought in the migrants’ province of origin, which means distant travel and additional costs.

The basket of services covered by a given public health insurance program varies across the Chinese territory. This basket depends on the level of funding, which in turn depends on local wealth. It leads to inequality in healthcare access from one county to the other [20,21]. With other policy measures as the zero-mark-up policy, public health insurance programs have contributed to alleviate the financial burden of healthcare for families. However, the reimbursement level is, so far, too low to provide a real shift in a patient’s pathway. The 2018 reform includes elevating the NCMS coverage to URBMI standards, but migrant workers are still under-covered for their healthcare needs [22].

Public health insurance schemes prioritize the reimbursement of severe and costly diseases over more frequent and less expensive care that also may require hospital admission. As a result, despite the implementation of public health insurance programs, spatial healthcare access is still mainly skewed towards admissions in public hospitals.

Mei was waiting for a consultation at the emergency department of The First Affiliated Hospital of Zhongshan University of Medical Science, Guangzhou.

“*I do not want to go to a community health center*. [*…*] *I want to have warranty of the medical staff’s quality. I feel more comfortable and secure to be here. They can perform so many different medical tests. Moreover, the results can be obtained very quickly. On my part, this is the service that I expect from a healthcare institute. Even if I have to wait for a visit, I save time because they can do a lot of medical stuff in the same location. Moreover, the medical staff is well trained*. [*…*]*Moreover, your medical information is easily shared between healthcare professionals.”*

Another difference between Hong Kong and China’s largest cities is how the health insurance market is organized. As I have previously explained, there is no public health insurance in Hong Kong. On the other hand, the PHI market is well structured. For primary healthcare, it can be based on the model of Health Maintenance Organizations, of Preferred Providers Organizations, or other types of managed care. Therefore, introducing e-health, as in U.S. managed care, will be achievable as soon as local market demand and legal regulations are set up to boost it.

In Mainland China, among a series of healthcare system reforms, the NDRC announced in March 2012 its clear intention to expand PHI [23]. A new regulation in 2014 enabled insurers, domestic and foreign, to invest and own more than one company in the same segment of the industry. Foreign companies (as well as domestic investors) may now own a stake in more than one insurance provider. In today’s China, the rise of PHI is supported by the emergence of an upper middle class and self-employed workers [24]. However, if this is a growing market, its size is still not sufficient to really play a role in the game of the spatial dimension of healthcare access.

Most recently, the government has encouraged employers to purchase PHI for their employees, in addition to UEBMI scheme benefits. The government has also offered tax incentives for employers and individuals that subscribe to a PHI in several of the largest cities.^15^

**Figure 4:**
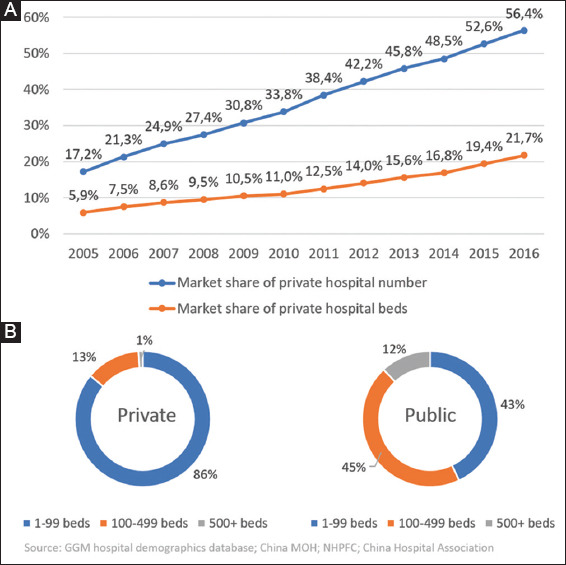
Public and private hospitals trend in China. (A) Source: Deng et al., 2018 [25]. (B) Source: https://www.asianhhm.com/healthcare-management/china-private-hospitals#:~:text=There%20are%20now%20about%2026%2C000,to%20hospital%20status%20every%20year

This deliberate policy to improve healthcare offers created the conditions for a spectacular increase in the number of private healthcare structures over the last decade. The number of hospitals has grown from 18,000 in 2003 to more than 33,000 today. Among these, the proportion of private hospitals is steadily increasing. It reached nearly two-thirds of the total number of hospitals. However, the size of public hospitals is usually much larger, be it in number of beds or admissions (Figure 4). In terms of the number of beds, they still account for more than three quarters of the total [25].

The government accelerated its support of e-health services and devices from the 2009-reform. With the 12^th^ 5-year plan, regarding the medical equipment industry, the MoC said, “the latest technologies in digitalization, internet, and cloud will be the key for electronics healthcare development.”

The MoH, in the Strategic Report on Healthy China 2020, planned a USD 9.8 billion budget to standardize IT system in major hospitals, building a public EMR system, and regional medical information platform. Indeed, IT systems were far from being standardized in major hospitals [26-28].

In addition, in 2019, the National Healthcare Security Administration introduced the Electronic Medical Insurance System. In May 2020, the National Health Commission (NHC) pushed provincial governments to establish their own online regulatory platforms to oversee and regulate individual online medical providers and to accelerate the market access of internet-based hospitals. In August 2020, the use of electronic licenses and certificates for medical institutions, nurses, and doctors was officially launched.

The guideline for Remote Medical Service from NHFPC, published in August 2014, allowed online service providers to offer medical suggestions, but so far, only healthcare institutions can provide remote medical treatment. In January 2015, telemedicine pilot programs have been launched in 5 provinces, amongst the most remote in China (Ningxia, Yunnan, Inner Mongolia, Guizhou, and Tibet). On September 2018, the PRC NHC promulgated three administrative measures to regulate telemedicine:

- Measures for the Administration of the Internet-Based Diagnosis and Treatment
- Measures for the Administration of the Internet Hospitals
- Measures for the Administration of Telemedicine.

These measures recognize the legitimacy of emerging internet-based healthcare services, as well as traditional telemedicine services (already defined by the existing regulations). In addition, the scope of the 2014 circular has been extended. From physician-to-physician consultations, it broadens the scope of telemedicine to include internet-based diagnosis and treatment provided by hospitals to their patients. It also includes internet hospitals that provide online diagnosis and treatment of common and chronic illnesses to all patients.

Today, a growing number of Tier 1 and AAA hospitals admit patients only after online pre-registration. It is done with a mobile phone app. Features included in apps such as WeChat include advanced functionalities, such as sharing blood test results or other diagnoses between medical staff and patients. According to many doctors, this system of registration has already changed the atmosphere at the hospital’s gate. A doctor from Beijing Anzhen Hospital, Nie Xiaolu explains^16:^ “*It used to be common to see more than 1,000 patients and family members queuing up in the hospital early in the morning*.” In the same hospital, the medical staff reports now “*the hospital space is quite quiet and empty*.” Annually, this hospital received 2.6 million outpatients.^17^

There are also a burgeoning number of apps proposing information-sharing systems and easy-to-use online platforms. These apps allow patients to access their personal calendars, local hospitals, and physicians, as well as help them make appointments within minutes. The services offered include the possibility to make payments using its mobile app and web portal.^18^

## 4. A Dynamic Perspective

So far, the two healthcare systems have been presented from a static point of view. The rapid growth of the elderly population has to be taken into account. It will be part of the equation for the transformation of any healthcare system. In Mainland China, in 2015 there were 5.9 million beds, of which more than 5.5 million were for the care of the elderly. In total, 2.8 million seniors are residents of an institution.^19^ In Hong Kong, the context is much more critical. In recent years, we have observed an increase in healthcare demand due to an ageing population with more medical needs [29]. How can the Hong Kong Hospital Authority financially support this increase? To be financed, the whole healthcare system needs to be sustainable.

### 4.1. The EMR

One of the key levers to adapt the system is the use of big data. In that regard, the EMR has been set up for years in Hong Kong. Hospitals under HA supervision have an electronic patient record system and they are capable of data sharing on a large scale. However, this health information is not used as a tool for reimbursement. As a consequence, the incentive in terms of health-related and medical data records is quite low.

So far, it is not relevant to compare a territory with a payment system based on this electronic health record and the largest cities in Mainland China, where such a system is under construction. The results will be a mix of the difference in the incentive to record and a difference in the performance, or hospital’s efficiency, without any way to disentangle these two mechanisms and to reveal each of them separately. The EMR use, though, can be associated to a DRG-based payment. This is a fee payment where each fee is set up as the average cost of the activity for the equivalent disease, diagnosis, co-morbidity and procedures (to a certain extent). The cost average is computed for the whole set of hospitals. In Hong Kong, the average cost could be computed from the set of hospitals under the supervision of HA. In the other largest Chinese cities, there are now different networks of hospitals using a common record EMR system, making such a payment system possible across each network. As a consequence, with the 2019 DRG reform, we may expect that in the near future, the admission price could be independent from the hospital but could depend on the network. For the patient, the advantage will be to foster medical price homogeneity over different locations.

In both Hong Kong and key cities in Mainland China, the way for patients to receive care includes inpatient admittance in hospital centers and outpatient follow-up with supervision from home, using digital tools as support. One example can be found with Type 2 diabetes and hypertension patients, where nurse-caregiver substitutes can be created with technology to support chronic disease self-care [30]. However, the medical staff caregiver substitutes go much further in Mainland China, where accessibility to a qualified doctor is at stake. The “soft” medical part of conventional in-hospital care can then be done remotely. Then, in such a framework, getting a standardized IT system will be secondary. Hospitals have already started to shift their care to the outpatient setting. As newly defined by law, a medical institution can choose to offer telemedicine services through its own medical team. As an alternative, it can also set up an affiliated internet hospital and invite external medical teams for telemedicine. Both arrangements require advance approval by local health authorities, and the medical professionals offering telemedicine must be licensed to practice in China.

The recent “Modern Hospital Administration System” reform aims at decreasing out-of-pocket expenditures and hospital length-of-stay. Dealing with patients remotely as much as possible is a way to reach this target.

However, it raises the issue of data sharing, interoperability, and analytics that will all have to improve in order for hospitals to realize the full benefits of using technology — not only to bolster outpatient care, but also to achieve value-based care results.

### 4.2. E-consultation

**Figure 5:**
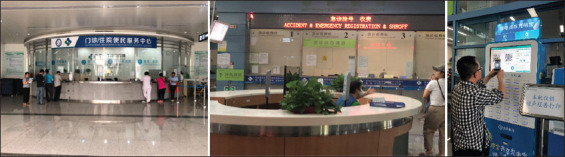
The situation in the Accident and Emergency Department, Hospital of Shenzhen at 2:30 pm on a weekday in May 2018.

The development of remote consultations is one of the avenues available to solve the shortage of high-level public hospitals in Mainland China (Figure 5). Highly qualified specialists in different medical disciplines can now remotely perform procedures that would have been impossible otherwise. Of course, one can argue that patients could have been transferred. However, sometimes shortening the waiting time to get a procedure saves lives or, at least, reduces the probability of complications. Moreover, transferring patients to another hospital is not so simple. On top of logistical issues, the unstable health status of the patients may prevent them from being transferred.

Furthermore, well-regulated remote consultations can be a way to deal with the increase in healthcare needs due to an ageing population. This solution is in its early days in Mainland China (mainly in several major urban centers) and even more so in Hong Kong. However, China is already the world’s third largest Med-Tech market. Observers expect a rapid increase in the years ahead. The Law on Promotion of Basic Medical and Health Care took effect in Mainland China in June 2020. It promotes medical information exchange, safety systems and telemedicine services.

### 4.3. Integrated model of online-offline healthcare

Moving beyond the traditional scheme, offering on one hand primary care, and on the other in-hospital care, China’s Internet giants are preparing to offer a complete array of healthcare services in which patient care will be provided remotely. Offline service will be a link in the patient pathways, and most of the time, the patient will be treated from her/his home.

Alibaba Group is one such Internet giant, initially specializing in online retail, as Amazon does in the United States. The group launched the “Future Hospital” program in 2014. This program aimed at consulting doctors online and ordering drugs (prescribed drugs as well as over-the-counter drugs) through the Internet. In spite of regulatory back and forth regarding over-the-counter drug sales, online pharmacies are now growing quickly. The health unit of JD.com Inc., another online retailer, and that of Alibaba are today among the leaders of this market.

AI is now a key component of the coming integrated model of online-offline healthcare. Alibaba recently introduced AI software that helps interpret CT scans. Tencent deployed Miying, which helps doctors detect early signs of cancer, and is now used in the hospitals of some of the largest cities in Mainland China. The search engine company Baidu scrapped its internet health care service that allowed patients to book doctor appointments through an app, in a bid to focus solely on AI.

**Figure 6:**
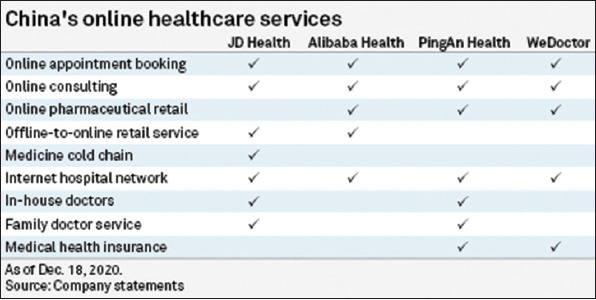
A coming integrated model of online-offline healthcare. Source: https://www.spglobal.com/marketintelligence/en/news-insights/latest-news-headlines/china-s-big-tech-to-continue-disruption-of-healthcare-sector-despite-crackdown-61610882

Lee and Seong [31] indicate that major hospitals are, at present, using AI-enabled systems to augment medical staff in patient diagnosis and treatment activities for a wide range of diseases. Going further, AI associated to cloud computing and a whole set of e-health options can lead to a transformation of the entire care service industry (Figure 6).

To summarize, the Internet and online healthcare are supposed to solve many issues. First, they enable people to overcome geographical obstacles to healthcare. Second, they allow people online access to qualified doctors and medical staff for e-consultations, as well as check or double-check on blood tests, and exploratory acts. Third, prescribed drugs, as well as OTC drugs, can be delivered at home in a few hours. Fourth, medical professionals can be assisted by IA, or IA chatbot can even be the entry to the healthcare patient pathway. Fifth, patient information can be stored and managed on a cloud and made available to healthcare professionals. Sixth, e-health devices and connected devices may help provide information to patients, routinely and in real time, to support care and prevent healthcare issues. In such a hypothetical context, the need for a strong and tight offline web of healthcare services would no longer be needed to achieve an efficient healthcare system. This will have an important impact on the spatial dimension of healthcare. In addition, the financial burden of healthcare access could be alleviated with such a system.^20^

## 5. Conclusion

The healthcare system in Hong Kong and several other major Chinese urban centers is a perfect illustration of the negative correlation between the concentration of healthcare centers providing high-quality healthcare and e-health. Through determinants of the inefficiency of each of the systems, the reader then observes the similarity of the questions at stake and of the answers obtained. This is the objective of developing the use of digital healthcare. The aim can be to foster the sharing of patient medical information between all medical staff involved; it can also be to deal with hospital congestion. It then contributes to ease of access via a virtual consultation. The offline medical consultation appears then as a second step of the patient’s pathway. In one word, the two systems seem to shift away from a two-step patient pathway, implemented with difficulties, to an easier-to-implement three-step pathway: e-consultation, offline medical consultation, and hospital admission where, at each step, the level of healthcare quality is judged acceptable. In this model system, where e-health is the first step and an integral part of the healthcare system, the concentration of healthcare centers appears to be a secondary issue, or more likely, irrelevant.

## Footnotes

1 Two sources: 1-The Legatum Institute, a London-based research institute released its annual global Prosperity Index, 2017; 2- Bloomberg introduced the Bloomberg Health-Efficiency Index, 2018.

2 United Nations Populations Division. World Population Prospects: 2019 Revision.

3 Tier 1 cities are the largest and wealthiest – often considered the megapolises of China.

4 See Milcent (2018a) for an extensive review of the Chinese Health care system.

5 Hong Kong’s food and Health Bureau, 2019. https://www.fhb.gov.hk/statistics/en/dha/dha_summary_report.htm#:~:text=Total%20health%20expenditure%20amounted%20to,GDP)%20during%20the%20same%20period.

6 Hospital Authority (2015). Hospital Authority Statistical Report 2014-2015. Retrieved from http://3w.ha.org.hk/haho/ho/stat/HASR1415_2.pdf

7 Lili was interviewed in January 2018 after her delivery at Princess Margaret hospital, Hong Kong, SAR.

8 See Figure 1, Hong Kong hospitals number

9 May’s mother was interviewed in March 2017 at Queen Mary hospital, Hong Kong, SAR.

10 Larina was interviewed in November 2017 after her discharge at Queen Mary hospital, Hong Kong, SAR.

11
http://hk.localiiz.com/public-or-private-a-comprehensive-guide-to-healthcare-in-hong-kong/#.WxTwPiOB124

12 Ministry of health of PRC, 2012. China Health Statistics Yearbook 2012. Beijing: Peking Union Medical College Press.

13 Deloitte analyses based on data from AHA annual survey, Medicare Cost Reports (via Truven Health Analytics).

14 Chou was interviewed in September 2017 at the emergency unit of Pekin University hospital, Beijing, China.

15 Ernst & Young. China Launches Pilot Individual Income Tax policy for Commercial Health Insurance Products; Ernst & Young LLP: London, UK, 2016.

16 No more long queues in China’s smart hospitals, China Daily, 8 Dec 2015

17 https://www.anzhen.org/Html/News/Articles/76.html accessed April 2021

18 McKinsey analysis, 2015 https://fr.slideshare.net/fle864/20150402-chic-mc-k-e-health-in-china-vf accessed September 2017 http://www.iamwire.com/2015/09/practos-chinese-counterpart-guahao-raises-394m-1-5b-valuation-led-hillhouse-goldman/123356 accessed September 2017 http://www.sramanamitra.com/2016/06/17/billion-dollar-unicorns-guahao-targets-chinas-healthcare/accessed September 2017

19 Source: National Bureau of Statistics of China, “Statistical Communiqué of the People’s Republic of China on the 2014 National Economic and Social Development”

20 In this paper, I do not deal with issues of data confidentiality and patient privacy.

## ANNEXE

**Annexe A:** Public charges – Eligible persons (for details on note N1, N2 and N5, see footnote 16)

Service	Fee/HK$
Accident and Emergency	$180 per attendance
Inpatient (acute general beds)	$75 admission fee, $120 per day ^N1,N2^
Inpatient (convalescent/rehabilitation, infirmary and psychiatric beds)	$100 per day ^N1,N2^
Specialist outpatient (including allied health clinic)	$135 for the 1st attendance, $80 per subsequent attendance, $15 per drug item ^N5^
General outpatient	$50 per attendance
Dressing or injection	$19 per attendance
Psychiatric day hospital	$60 per attendance
Geriatric day hospital	$60 per attendance
Rehabilitation day hospital	$55 per attendance
Day procedure and treatment at Clinical Oncology Clinic or Renal Clinic	$96 per attendanc
Day procedure and treatment in ambulatory facility $195 per attendance
Community nursing service (general)	$80 per visit
Community nursing service (psychiatric)	Free
Community allied health service	$80 per visit

Source: http://www.ha.org.hk/visitor/ha_visitor_index.asp?Parent_ID=10044&Content_ID=10045&Ver=HTML, accessed 2 June 2018. For details, please refer to the Gazette –Public Charges – Eligible Persons.
